# Distinguishing synchronous and time-varying synergies using point process interval statistics: motor primitives in frog and rat

**DOI:** 10.3389/fncom.2013.00052

**Published:** 2013-05-09

**Authors:** Corey B. Hart, Simon F. Giszter

**Affiliations:** ^1^Neurobiology and Anatomy, Drexel University College of MedicinePhiladelphia, PA, USA; ^2^Lockheed Martin CorporationPhiladelphia, PA, USA

**Keywords:** synergy, primitives, synchronous synergy, time-varying synergy, point process

## Abstract

We present and apply a method that uses point process statistics to discriminate the forms of synergies in motor pattern data, prior to explicit synergy extraction. The method uses electromyogram (EMG) pulse peak timing or onset timing. Peak timing is preferable in complex patterns where pulse onsets may be overlapping. An interval statistic derived from the point processes of EMG peak timings distinguishes time-varying synergies from synchronous synergies (SS). Model data shows that the statistic is robust for most conditions. Its application to both frog hindlimb EMG and rat locomotion hindlimb EMG show data from these preparations is clearly most consistent with synchronous synergy models (*p* < 0.001). Additional direct tests of pulse and interval relations in frog data further bolster the support for synchronous synergy mechanisms in these data. Our method and analyses support separated control of rhythm and pattern of motor primitives, with the low level execution primitives comprising pulsed SS in both frog and rat, and both episodic and rhythmic behaviors.

## Introduction

The efficient control of an organism's motor architecture poses significant difficulties for the central nervous system. In particular, control of the limbs is an ill-posed problem: too many possible solutions are available to perform a particular motion for the nervous system to find the correct combinations of muscles in a timely manner. As a solution to this problem, a variety of modular control strategies have been presented (Giszter et al., 1989, 1991, 1992, 1993, 2007, 2010a,b; Mussa-Ivaldi et al., 1990, 1994; Bizzi et al., 1991, 1992; Mussa-Ivaldi, 1992; Mussa-Ivaldi and Giszter, 1992; Giszter and Kargo, 2000, 2002; Kargo and Giszter, 2000a,b; Mussa-Ivaldi and Bizzi, 2000; Mussa-Ivaldi and Solla, 2004; Cheung et al., 2005, 2009; d'Avella and Bizzi, 2005; d'Avella et al., 2006; Tresch et al., 2006; Bizzi et al., 2008). Modular control of motor structures reduces the number of independent points of control for the system and therefore reduces the number of degrees of freedom available in the execution of a movement.

### Types of modularity

Specifying that the motor system employs a modular control scheme does not, in the end, tell us very much. There are many different kinds of motor modularity. For example, some groups have found evidence of “kinematic modularity” or regular, repeated structure in movements or the planning level of movements (Viviani and Terzuolo, 1982; Hogan, 1984; Sosnik et al., 2004; Chiovetto et al., 2010; Omlor and Giese, 2011). In contrast, and perhaps as complement to kinematic modularity, there is execution modularity, or modular organization in the control mechanisms underlying performance of a particular task or set of tasks. Many examples of execution modularity have been reported in recent years and include such examples as central pattern generators (Grillner, 2006), half center oscillator models, blends (Stein et al., 1986; Stein, 1989), motor primitives (Giszter et al., 1991, 1993; Hart and Giszter, 2004, 2010), and time-varying synergies (d'Avella et al., 2006).

We are interested in examining two of these forms of execution modularity in detail. The first group, synchronous synergies (SS) (Hart and Giszter, 2004, 2010; Kargo et al., 2010), are built from synergistic groups of muscles activated with a fixed time course. Work done, both in our lab and several others, supports the observation that most movements generated by an unconstrained frog can be represented as the summation of multiple motor primitive style elements (Bizzi et al., 1991; Giszter et al., 1992; Mussa-Ivaldi and Giszter, 1992; Giszter et al., 1993; Mussa-Ivaldi and Bizzi, 2000). However, some researchers have advanced time-varying synergies an alternative to the motor primitive model (d'Avella et al., 2006). In this model, temporally coordinated (but not necessarily synchronous) drives are supplied to groups of muscles. These time-varying synergies are thought to form sequence units. Kinematic strokes form modules, and the time-varying synergy (TVS) might be thought to correspond to such higher order task units. Such time-varying drives, as formulated in theory, may be dilated uniformly across their temporal duration, as required by the task. In the TVS model described above, there is a strong connection between the duration of a sequence of a pulses on several muscles and the temporal widths of the pulses supplied to those muscles, i.e., uniform temporal scaling across the entire motor compositional unit.

To distinguish these two frameworks: “spatial/synchronous muscle synergies” and time-varying synergies we have examined how these models constrain the onset and peak timings of muscle activity viewed as point processes. To do this we will construct models of point processes reflecting both synchronous and TVS schemes. We show that distinct point process statistics arise. We plan to test the hypothesis that real electromyogram (EMG) activity recorded in frogs resembles activity that one would expect to see generated by a synchronous synergy model of motor production, rather than a TVS model of motor production and examine the degree to which statistics computed on real EMG data from 10 hindlimbs of the bullfrog resembles the statistics computed on each model.

There have been many metrics and procedures developed for quantifying the level of synchronous activity between sets of point processes. Many of these statistics have attempted to quantify synchronicity in terms of metrics computed between pairs of spike trains, such as cross corellograms and joint peristimulus time histograms (Ellaway and Murthy, 1985; Adams et al., 1989; Datta and Stephens, 1990; Nordstrom et al., 1990, 1992; Bremner et al., 1991a,b; Datta et al., 1991; Ushiba et al., 2002), although most of these metrics show significant sensitivity to the density of events within measured intervals (Bremner et al., 1991a,b; Kim et al., 2001). Higher order synchronization (between three timestamps) has been examined and quantified using such tools as the snowflake plot (Perkel et al., 1975; Czanner et al., 2005). However, statistics on synchronicity for time series of arbitrary dimensionality are lacking. Since the number of muscles participating in synergies is not necessarily fixed, this lack represents a problem for comparison of time series representing the activation of differing types of multi-muscle synergies. In light of these difficulties, in order to make comparisons between such processes, we have developed a metric that shows sensitivity to the degree of onset or peak synchronization over a wide range of parameters. We have performed extensive testing of this statistic using data from models of both synchronous motor synergy strategies and TVS strategies. We then apply this statistic to frog motor pattern data.

## Methods

All experimental data from frogs and rats used in this paper for exposition was obtained under strict compliance with USDA and PHS guidelines, and with full oversight of the Drexel University College of Medicine IACUC.

### Data construction

Both real data and modeled data were used and tested in the developed type of discriminant analysis.

#### Real EMG data collection

Real EMG data were derived from 10 spinalized frogs, with recording electrodes in 10 hindlimb muscles. (RA, RI, AD, SM, GL, VI, BI, SA, VE, ST). Frogs were anaesthetized, spinalized, and decerebrated. Ball electrodes, constructed as in (Hart and Giszter, 2004) were implanted in these 10 muscles of the hindlimb. After at least a day of recovery, EMG recordings were made during a variety of frog behaviors at 2 kHz. EMGs were recorded using a Cerebus 128-channel data acquisition system (Blackrock Microsystems) and saved to a file. Files were imported into the MATLAB™ programming environment for analysis. Imported EMG data were rectified, smoothed using an 80 point, triangular window, moving average filter, and down sampled to 250 Hz. The data were then saved to a MATLAB MAT-file and retained for later analysis.

***Basic model data generation parameters.*** The basic model was defined as a renewal process, constructed on intervals of variable duration, ranging between 90 and 250 ms. Every Δ = 90–250 ms, an interval value was drawn from a Poisson interval distribution (exponential), and a pulse or sequence of pulses was placed at the cumulative sum of intervals up to that draw. The distribution of intervals constructed in this manner was approximately Poisson, with a maximum interval cutoff below 2 × Δ ms (because every interval is populated with a draw). There was also some distortion away from true Poisson at the longer time scales, due to the fact that the Poisson process “reboots” at the edge of every Δ ms interval. We tested and compared several different point process distributions. However, these did not affect our basic discrimination findings and here we focus entirely on the Poisson process results.

### Discriminant method

We examined the effect of varying several synergy parameters on the subsequent discriminability of synchronous and time-varying synergies from one another using point process model statistics, and to distinguish the sum of time intervals between local maxima in each type of time series. These parameters were the density of synergies on an interval (ρ), the number of muscles per synergy (σ) and the number of simultaneous muscles in TVS models (η). We do not assume any *a priori* knowledge of σ in real-world data, although we do know that the majority of primitives examined in our work generally contribute significantly to between ~2 and 4 muscles (Hart and Giszter, 2004), and because synergistic groupings with more or less muscles are possible.

Two distributions were used to model σ. The first distribution was sharply peaked and σ was permitted to vary between 2 and 10 muscles, with a maximum likelihood occurring at around 3 muscles. A second flat distribution, in which all values of σ are equiprobable, was also used. Other parameters, such as pulse amplitude were not expected to have a significant effect, based on the structure of the onset or peak-picking algorithm (the peak detection algorithm identifies all peaks above the noise threshold based on their resemblance to a template peaked waveform) but were examined as well. For each model run, the total number of synergies was selected, at random, from a Gaussian distribution with a mean and median of ~4 synergies/run, omitting negative results.

*Synchronous Synergy Model Construction (SS model)* Modeled data were constructed using the two different models (synchronous vs. time-varying), and used to act as the main poles of comparison for real data in this discrimination task.

**Figure 1:**
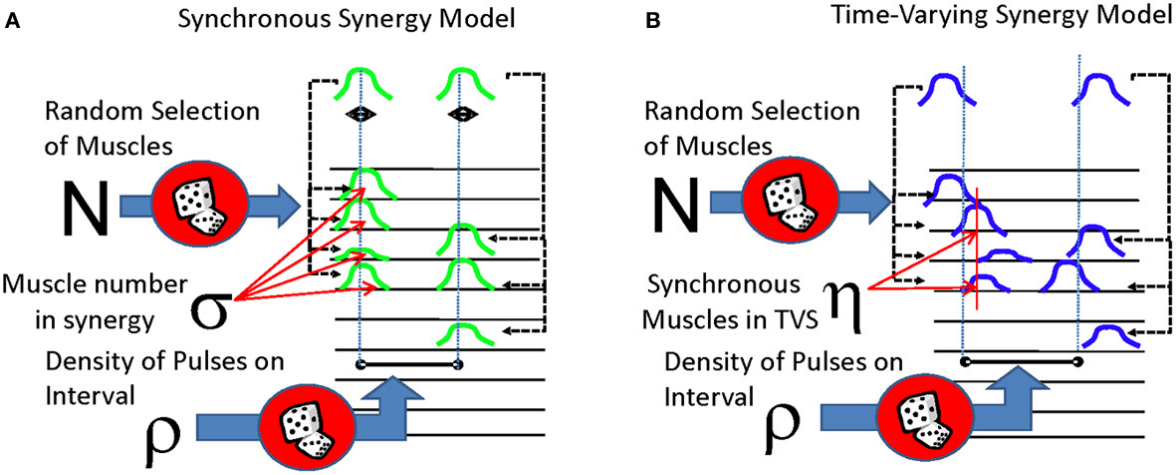
**Two forms of synergistic muscle activity compared in this analysis. (A)** Synchronous synergy model. Pulses are multiplexed to several muscles to several muscles, with tight coordination of temporal activity of each pulse. **(B)** Time-varying synergy model. Pulses are sent to several muscles with some time delay between them, and the entire set of pulses and delays may be dilated or contracted as necessary. In both models, the delay between different synergys is drawn from an exponential distribution (or in a few cases, from a uniform distribution).

The first model (Figure 1A) represented an idealization of SS or motor primitives. This model was constituted of several simultaneous pulses delivered to a subset of channels (“muscles”) that are multiplied by a channel-specific gain. The EMG was constructed on intervals of 500 ms and the density (ρ) of possible synergies delivered on a single interval was varied between 0 and 5. Each simulated EMG would be constructed of several distinct synergies. The number of distinct synergies to be used in the entire constructed data set was selected and allowed to vary stochastically between different realizations, with care being taken not to include any duplicate synergies. (As an example of this constraint, if there were 8 muscles in one synergy, then only 2 non-identical and non-overlapping or disjoint synergies could be drawn, one synergy with 8 muscles and the second synergy with the remaining 2 muscles). We then created and drew from a distribution representing the number of possible muscles (σ) participating in each of these synergies. As mentioned in the preceding section, this distribution was permitted to be either a peaked function (reaching a maximum between 2 and 4 muscles per primitive, consistent with observed data) or a flat function where primitive muscle membership numbers were all equiprobable. For each synergy, we then randomly selected “muscles” from the 10 available muscles.

SS synergies were modeled as occurring in episodic motor patterns. After a time of occurrence of the first SS synergy (the episode onset) was chosen, other SS synergies were positioned to occur following. To accomplish this, the (randomly selected) following SS synergies in the motor pattern were assigned random onset times on the 250 ms interval following a first “seed” synergy event of the episode. This created a point process representation of motor pattern under the SS model. A continuous signal was then constructed for each synergy's component muscles, and then these were combined to obtain the different activity patterns on each virtual EMG channel. More specifically, we proceeded as follows: A gaussian pulse forming the “seed synergy” with a time course of around 300 ms was placed at each of the point process sampled motor pattern event times. Each synergy pulse occurring on the interval was positioned shifted from the “seed synergy event” by a small amount, drawn from an exponential distribution, with a mean value of 250 ms and a median of 153 ms. Given that several synergies may in this way be invoked on the same interval, it is thus possible that there may be several overlapping pulses in a motor pattern “event” interval. In this case, the amplitudes of each pulse of an individual muscle's activity occurring in overlapping synergies were summed together. After the synergies were positioned in time the EMG signals were next calculated in this way. White noise was then added to all EMG channels of the model, with a maximum noise amplitude of 5% the maximum value of the clean signal. Amplitudes of each Synergy pulse were assumed to be either the same, or to be drawn from a normal distribution, with a mean value of 0.5, and truncated at amplitude values of 0 and 1. Amplitude effects on final results were negligible.

#### Time-varying synergy model construction (TVS model)

The second model (Figure 1B), used in this analysis was constructed to simulate various instantiations of a TVS model of motor control. A single pulse event was created and placed on a 250 ms interval at a randomly drawn time. Delays for the remaining pulse events from the drawn time ranged from −100 to 100 ms and were sampled from an exponential distribution with a mean of 100 ms (the sign of the delay was selected randomly). These shifted pulse events were then delivered to a subset of channels (“muscles”) that were multiplied by a channel-specific gain. A small number of such TVS were then randomly selected to be a set used in an individual realization, by repeating a randomly selected member of the set at the (Poisson distributed) synergy event times. Each such constructed synergy occurrence was scaled in time in its overall duration by a factor that was drawn from an exponential distribution with a mean of 2. This created a point process representation of motor pattern variations under the TVS model. Apart from these changes, the TVS model continuous signal representation was then constructed as in the SS case. TVS models could thus range from models in which there were no synchronous drives delivered to any muscles, to models in which synchronous drives are supplied to many muscles in the TVS, while other muscles in a synergy are asynchronously activated. This latter variant could in principle be thought of as a TVS containing within it some number of SS.

#### Discriminant analysis of real and model data

Simulated data from both TVS and SS models were compared with rectified, filtered, and downsampled examples of real data from frogs.

**Figure 2:**
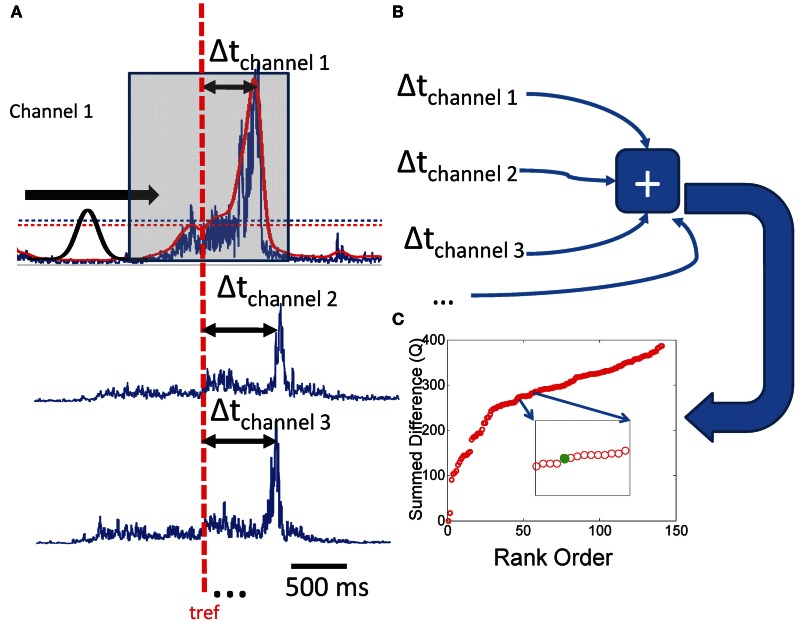
**Calculation of the Q statistic. (A)** On an interval ranging from −250 to 250 ms, we identify peaks in a rectified and smoothed set of EMG waveforms by sliding a gaussian waveform along the intervals and identifying points of maximum correlation with amplitudes larger than a rejection amplitude (more than 2 sds the mean EMG activity). Peak times are subtracted from a randomly drawn reference time from each interval, and the **(B)** absolute differences are summed over that interval. **(C)** The same procedure is performed for all such non-overlapping intervals on the data set. The resultant Q values are rank ordered for the purpose of comparing distributions from different sets of data.

Peak/Onset times were extracted from each channel of real or simulated EMG by thresholding the EMG to 2 standard deviations above its mean value and identifying all points which exceeded this value. We then used a sliding gaussian pulse of duration 250 ms to discriminate values over the predefined threshold which represented peaks in the recorded data. We chose this peak width as it most closely reflected the dominant time scale observed in EMG recordings in bullfrog (Hart and Giszter, 2004). However, this peak discrimination process was simply to subject both real and artifically created data to a similar workflow and any errors introduced in peak or onset selection. Peaks were identified as those points over the amplitude threshold where the correlation with the sliding Gaussian pulse is more than 2 standard deviations over the mean correlation value (Figure 2A). This criterion nearly always (>95% of the time) found the correct local maximum in a sequence of data. We then chose the times taken from one chosen sample channel of EMG as “reference times” to be used in the ensuing analysis. The times of peak occurrence on other channels were identified via the thresholding algorithm described above and the difference from the reference times were calculated on each successive 250 point time window defined around each reference time. Peak time differences were rank ordered within each window and the mean time difference on the intervals were calculated.

A cumulative statistic Q was then created for the time window by summing time differences (Figures 2B,C) as follows:

(1) $$Q=\frac{\sum_{t_{\text{ref}}}\sum_{t_{i}}\left(|t_{i}-t_{\text{ref}}|\right)\times \Theta (|t_{i}-t_{\text{ref}}|)}{N}$$

where Θ is a step function with value 1 when its argument is <250, and 0 otherwise. *t*_*i*_ is the time of a peak in the window, *t*_ref_ is the reference time, and *N* is the total number of pulse events in the analysis window.

The procedure for calculating Q was then repeated for the next time window in the record defined by the reference channel and reference times. We calculated Q values for each segment of the real data files, as well as each segment of both model implementations. This analysis was carried out on all runs of each model. Reference channels used were randomly assigned. In some instances all channels were tested as reference and compared. Choice of reference had little effect on statistics. This statistic, Q, captures the deviation of the peak clustering in SS and TVS patterns from that observed or expected in an unconstrained random distribution of pulse times, which was constrained to neither SS or TVS structure. The composite interval structures captured in the measure Q, will deviate from predictions based on uncorrelated point process assumptions, due to the correlations and constraints on intervals imposed by synergy structures. For example, the expectation of interval differences in unconstrained Poisson processes will be that they effectively represent the result of a Poisson process of higher rate, reflecting the independent constituent process rates. In contrast, SS constraints enforce short intervals in the joint process, and TVS enforce longer intervals, and also short intervals to the extent that SS are found within the TVS sequences.

#### Discriminant scaling analysis of real data

To compare models and data we used the cumulative distributions of the statistic Q. Given the likelihood that connected peaks in TVS data will be farther apart than in SS data, we expected that data consistent with a TVS model will have larger values of this statistic than data consistent with SS models. Given a set of randomly drawn reference times, and for any distribution of pulses on a given interval, the maximum likelihood (ML) of the absolute value of the difference between Q values computed from a set of reference times and the distribution of pulses will be non-zero. If data is described perfectly by a SS model, Q-values will cluster around the ML value for this model, more or less normally, by the central limit theorem. Q-values from data better described by a TVS model will cluster around the ML Q-value for TV synergies. Taking the difference between Q values for each model (Qss, Qtvs) and the real data (Qreal) we arrive at two error distributions, both approximately normal.

We further anticipated that by rescaling (i.e., dilating) small time differences in EMG peak times in SS models, we will eventually obtain Q-statistics characteristic of, or similar to a TVS model, or a non-SS random pulse pattern.

Rescaling of synergies was done in constructed data as follows. During construction of simulated data sets, time-varying synergies were generated according to the procedure outlined above and were then rescaled by a variable factor. Each synergy was scaled independently before adding it to the data. Consequently, synergies consisting of only a single muscle were not shifted in time, nor was the stochastic rate at which synergies were generated scaled, leaving the length of the time series intact. Constructed SS were scaled in an analogous fashion, although in this case the rescaling factor was applied to the small temporal jitter between pulses in a primitive (See previous section for details of SS contruction).

To scale real data for comparison, we first identified likely synergies by identifying near-synchronous pulses in different EMG channels. Any pulses with a time difference of less than 5 ms were selected as potential SS and retained for dilation and statistical analysis. Those collections of pulses that appeared more often than expected due to random chance (2 SD > average frequency of occurrence calculated from 50 shuffles of time indexes of all peaks detected in a record) were identified as “synchronous synergies” for the purpose of this analysis. The small jitter between the pulses in each such synergy were then scaled as above. Scaling was relative to the mean midpoint time for each synergy/collection of pulses, so early pulses were shifted backward and late pulses forward.

As stretch of intervals outside of 250 ms is impossible in our formulation i.e., it invalidates the interval for the analysis (it will push timestamps into next window), it follows that, dilation should not significantly move the Q difference distribution of TVS data. In contrast, dilation of the SS model data was expected to yield cumulative Q values that were eventually more statistically similar to the original TVS Q values computed. Assuming real data is described by the SS model, subjecting the data to this procedure should yield Q distributions that are similar to TVS distributions. If real data is better described by the TVS model, one would expect little change in cumulative Q upon performing the dilation operation. With this in mind, we took the timestamps associated with peak times in real data and scaled the differences in timestamps over scale factors that ranged between x2 to x20 and calculated Q values at each scale-step. Scaling the differences in timestamps and scaling the underlying waveforms made no difference, as the peak picking algorithm identifies the same peak for a pulse, regardless of its width. Differences between the median Q at each scale and the original unscaled median Q-value were retained.

#### Are primitive timing and dynamics related?

We also used more data-driven techniques to further assess the likelihood that synergies in the bullfrog consisted of SS style constructions, which has been our working hypothesis in prior research. To do this, we performed two analyses on real frog EMG data. In keeping with the Gottlieb work showing triphasic activation of muscle groups during movements (Gottlieb, 1998), we examined timing relationships within triplets of pulses extracted from frog EMG data. We first performed regressions of pulse widths for each pulse in a triplet against both the pulse widths of the other members of that triplet, and against the time delay between pulse 1 and pulse 2, as well as between pulse 2 and pulse 3. In order to assess the main sources of variance to the resultant regression coefficients, we then performed a principal components analysis on these coefficients, and generated projection biplots to assess the relative significance of the contribution of each component. Additionally, we took the entire sequence of pulse widths and pulse time differences, treated each of these variables as covariates for an independent component analysis (ICA) in order to assess the relative independence of each of the variables. Additional details on this analysis are provided in the Results section below.

## Results

### Difference of Q statistic for SS and TVS model data

**Figure 3:**
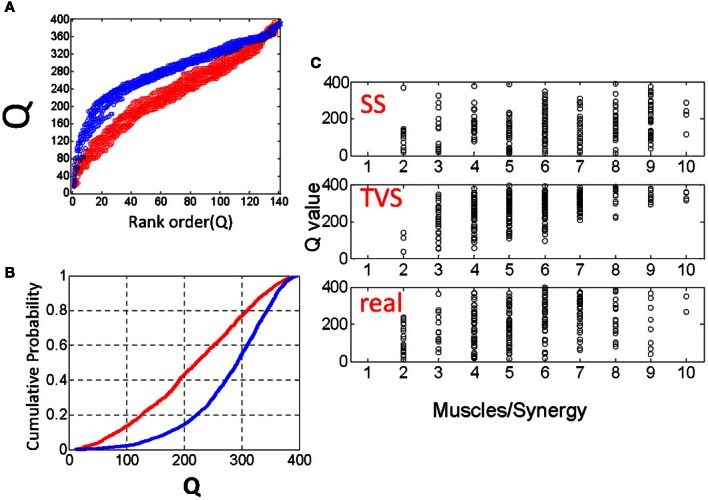
**(A)** Comparison of rank ordered Q statistics from five runs of the basic TVS model (blue) (ρ = peaked distribution, σ = 3, η = 1) and five runs of the basic SS model. **(B)** cumulative probability of the same Q distributions. **(C)** Comparison of the effect of ρ on local Q statistics for SS models, TVS models, and real data. Note that as ρ increases, Q statistics tend toward higher for TVS models than for real or SS constructed model data.

We constructed 25 time series for each parameter set as above (Poisson interval distributed events) and calculated Q values on simulated TVS and simulated SS models for each sampling on these series. For a single draw from a distribution with six components and an η = 1 and a ρ = 1/90 we found that the Q distributions were easily distinguished from one another. The rank ordering of Q values from each SS and TVS distribution (Figure 3A), or the cumulative probabilities for each distribution (Figure 3B) were clearly and cleanly distinguishable from each other. Additionally, we compared Q statistics from the distribution of uniformly distributed synergy events (see above) with those calculated using the Poisson interval distribution, for each of the chosen sets of parameter values. Q statistics could not be discriminated between the uniform and Poisson generators using paired *t*-tests (comparison of TVS statistics: *p* = 0.34, SS statistics *p* = 0.75). Lowering ρ to 1/250 did not appreciably alter the situation.

### Effects of different parameters

As previously mentioned, we recognized that varying the parameters σ, ρ, and η has the potential to alter our ability to discriminate the outcomes of these different models. There are several different parameters that must be examined for their effect on the discriminability of TVS and SS models. First, the number of synergies must be considered. Secondly, each synergy may be constituted by a variable number of muscles. The distribution of the number of muscles in a synergy may also have an effect on discriminability. Q values calculated on two muscle synergies will tend toward smaller summed differences than in five muscle synergies by nature of the number of items measured and summed. This aspect of the statistic is unavoidable. Finally, the number of synergies on each interval used to calculate a Q value can impact the value of the Q parameter as well.

The number of synergies on an interval can have a drastic impact on the value of Q, as can the form of the TVS synergy itself. Therefore, we examined each 250 ms interval of our constructed data, and looked at the number of pulses on that interval.

### Effect of varying σ

For small values of σ (the number of muscles per synergy), we find that the TVS, SS and real local Q distributions are very hard to discriminate. Q values that are very similar persist over all three models for a range of ρ values (Figure 3C), but begin to clearly diverge at a σ value of five or six muscles per primitive. For higher σ values, TVS and SS Q values are easily discriminated from one another. Real values, and SS synergies are much more similar than real and TVS values. The reason for this is easy to see. At small values of σ, only one or two muscles participate in a given synergy. Thus it becomes more difficult to say whether a given synergy is time-varying or not. At larger values, there are many pulses on an interval. For TVS, many of these pulses will be separated by significant time delays, resulting in a higher Q statistic. For SS, the pulses will be more tightly coordinated, yielding a lower Q statistic.

### Effect of varying ρ and η

Because σ (the number of muscles per synergy) tends to be around three or four muscles for both SS and TVS models as described in the literature and since σ and η (the number of simultaneous muscles in a TVS model) are going to be somewhat coupled, we chose to examine the effect on model discriminability of varying ρ and η simultaneously.

For our purposes, we are classifying as “synchronous” only those synergy models in which all muscle activations in a given synergy are simultaneous (within some error δ). Synergy models containing two or more out of phase muscle activations (with the remainder occurring at variable times) will be considered, for the purposes of this study, as “time-varying.”

**Figure 4:**
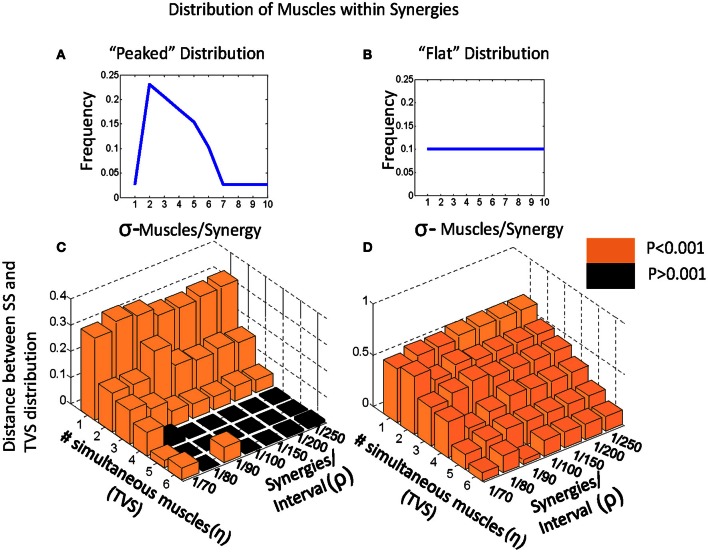
**Varying model parameters alters discriminability of models. (A)** A sharply peaked distribution. **(B)** A flat synergy distribution. **(C)** The peaked distribution resulted in easy discrimination of TVS models from SS models at an alpha level of 0.001 for up to three simultaneous synergies in TVS models. **(D)** The flat synergy distribution results in discrimination of TVS models from SS models for all values of ρ (density of synergies on an interval) and all η (the number of simultaneous muscles in a TVS).

Given this classification, we chose to examine how varying the number of simultaneous muscles in a TVS model interacts with the density of primitives on a 200 ms interval to impact the discriminability of TVS and SS models. We did this by identifying the points at which TVS and SS distributions were maximally discriminable, a measure which coincides with the Kolmogorov-Smirnov (KS) statistic. KS statistics for the discriminability of TVS and SS models, as a function of these variables are shown in Figure 4. TVS and SS models were constructed using both peaked (Figure 4A) and flat (Figure 4B) muscle/synergy distributions. As can be seen, for the peaked distribution, KS statistics were significant for a wide range of combinations of parameters. Further, the number of simultaneous muscles within a synergy did not appear to significantly impact the significance of these differences even at the lowest synergy density. For the peaked distributions, the Q statistic failed to distinguish TVS and SS models for high numbers of muscles (>4) in combinations that occurred only rarely on an interval (black floor in Figure 4C). Expanding the interval, or increasing the data set, might abolish this dead zone of discrimination. For the flat distribution of primitives, for all combinations of these variables, TVS and SS models were easily discriminable across all tested conditions.

### Distance between real data and SS data vs. real data and TVS data

**Figure 5:**
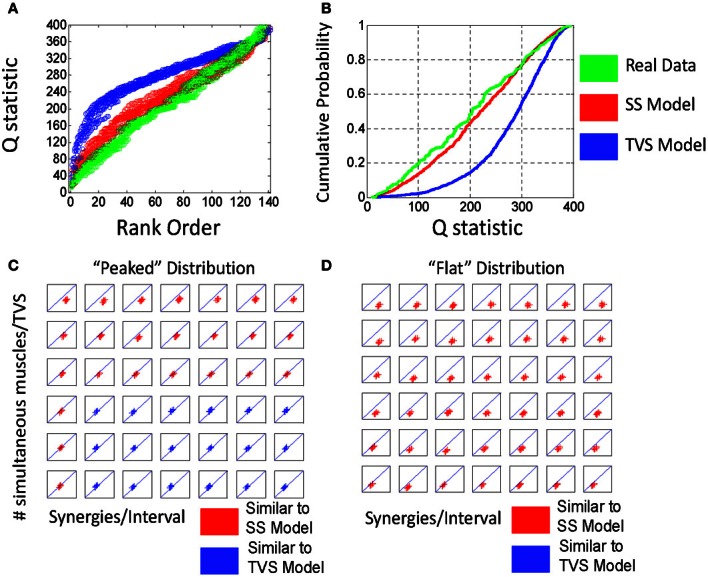
**Q statistically classifies real from EMG data as a synchronous synergy strategy for range of discriminability of the statistic.** Linear discriminant separates Qss-Qreal (y axis) and Qtvs-Qreal (x axis). **(A)** In data drawn from the peaked distribution. **(B)** Cumulative probability shows TVS/SS distinction. **(C)** For peaked distribution (see Figure 4A) we find that for a range of discriminable parameters (see Figure 4A) plotted points are less than unity, indicating Qss-Qreal < Qtvs-Qreal. All parameter pairs ρ and η show Qss-Qreal < Qtvs-Qreal. **(D)** For flat distribution (see Figure 4B) all parameter pairs ρ and η Qss-Qreal < Qtvs-Qreal. In both cases, this is consistent with a model where EMG activity is generated via an SS-type strategy.

We next calculated Q values for five records of real EMG activity (see methods). We then compared the middle of this Q distribution (Qreal) to the middle of Q distributions calculated for SS (Qss) and TVS (Qtvs) models at each of the parameter values examined above. We plotted the distance between Qreal and Qss against that between Qreal and Qtvs. Note that where the two distributions are broadly discriminable, either for synergy densities drawn from a peaked distribution (Figure 5A) or a flat distribution (Figure 5B) the distances between Qreal and Qss were smaller than Qreal and Qtvs (Figures 5C,D). Further, the real data is found ***beyond*** the model SS cumulative curve, rather than between the SS and TVS curves. The real data thus exhibited stronger SS statistics than our artificially created data. Accordingly, the cumulative distributions support the idea that the SS hypothesis for real data is likely still stronger than the *p* < 0.001 calculated for the artificial data. However, we limit our assessment to this value here.

### Effects of forcing dilation on data and Q statistics

**Figure 6:**
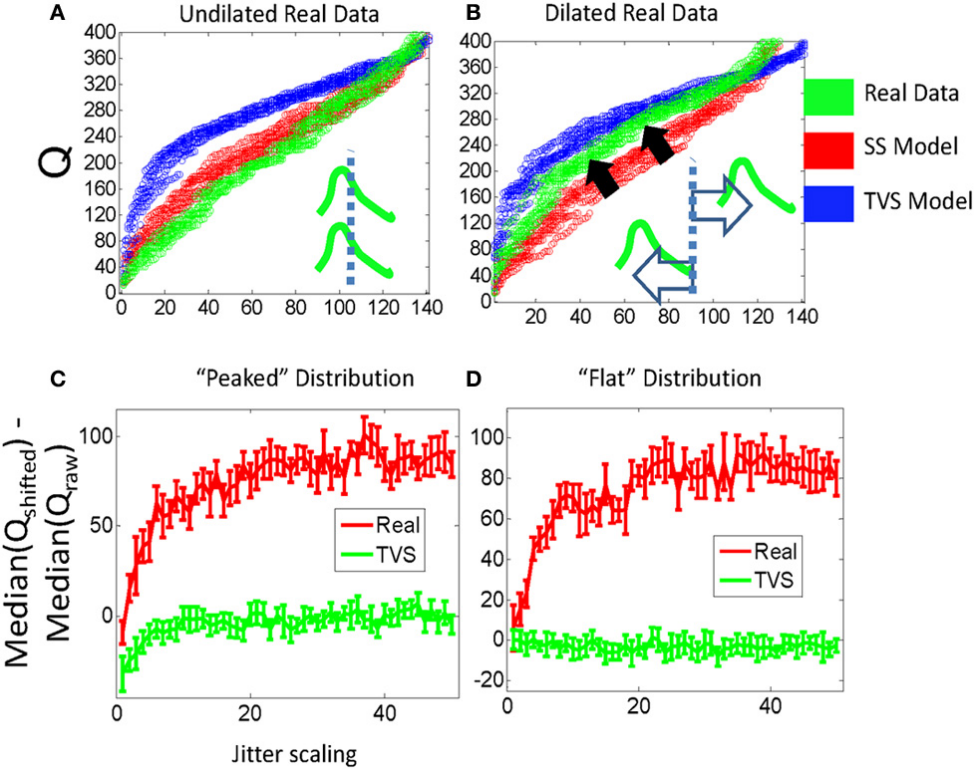
**Stretching the interval between jittered pulses causes real data that resembles SS model data to begin to resemble data generated by a TVS strategy, for a fixed analysis window. (A)** The Q statistic distribution of real data (green, left) which is clearly SS in form, is moved toward the Q statistic distribution of the TVS model data when intervals between real data are scaled linearly with an unscaled analysis window **(B)**. **(C)** Peaked σ-distribution: comparing the difference between scaled Q stats of real data and their unscaled values with scaled TVS Q values and the unscaled Q values implies real data is clustered more tightly around particular time scales (i.e., there is more room to scale intervals within the window before a timestamp is forced into the next counting interval and drops from the statistic) compared to TVS generated data. **(D)** Flat σ-distribution: note the nearly identical performance to that in **(C)**.

The construction of the Q statistic provides an additional test for the presence of SS synergies, based on a dilation manipulation of the synergies' time scale within the analysis window. The analogy is to using a higher powered microscope field, the intervals are dilated but some fall out of the analysis window field of view. If we assume that a particular distribution of pulses were constituted primarily of time-varying synergies with a maximum time scale of less than 200 ms (reasonable for frogs given that most movements are executed in under a half second), then finding the time of each pulse with respect to some arbitrary reference time on each interval and then scaling these times by a constant dilation within each interval should not impact the Q statistic significantly. In pure TVS synergies lacking SS components, or non-synergy processes, this scaling would simply push a few of the pulses out of the analysis window interval for computing Q values. So the Q value before dilation minus the Q value computed after dilation should often be near zero. In contrast, for a SS model, it should be possible to dilate all the short pulse times (with respect to a reference time) while keeping them all in the analysis window. As short intervals are highly associated with SS synergies (e.g., Krouchev et al., 2006; Markin et al., 2012)—dilation in the analysis window affects SS quite a lot, first altering Q statistics, before the Q statistics plateau. The statistic will plateau at the point at which pulses are pushed into the next computational interval). A new statistical measure then is obtained from the difference of the Q statistic after the dilation minus that of the unaltered data. This new difference in Q statistic should rise to a plateau at a particular scale value. We found that the dilation of real data time intervals does tend to push the Q statistic for dilated data toward the Q distributions for TVS data, and obviously this is captured in the difference. This is shown to be the case in Figure 6A. As the real data is dilated (Figure 6B), the cumulative distribution moves from its position beyond the synthetic SS model data, and crosses over to lie between the SS and TVS cumulative distributions. Furthermore (Figures 6C,D), the difference between dilated and unaltered data Q statistics in model data is close to zero for TVS synergies (tQ cannot be significantly altered as the analysis intervals are dilated) but increases significantly for real data or SS model synergies. This observation holds true for both peaked and flat synergy density distribution models.

### Connection between pulse timing and scaling of muscle activation

**Figure 7:**
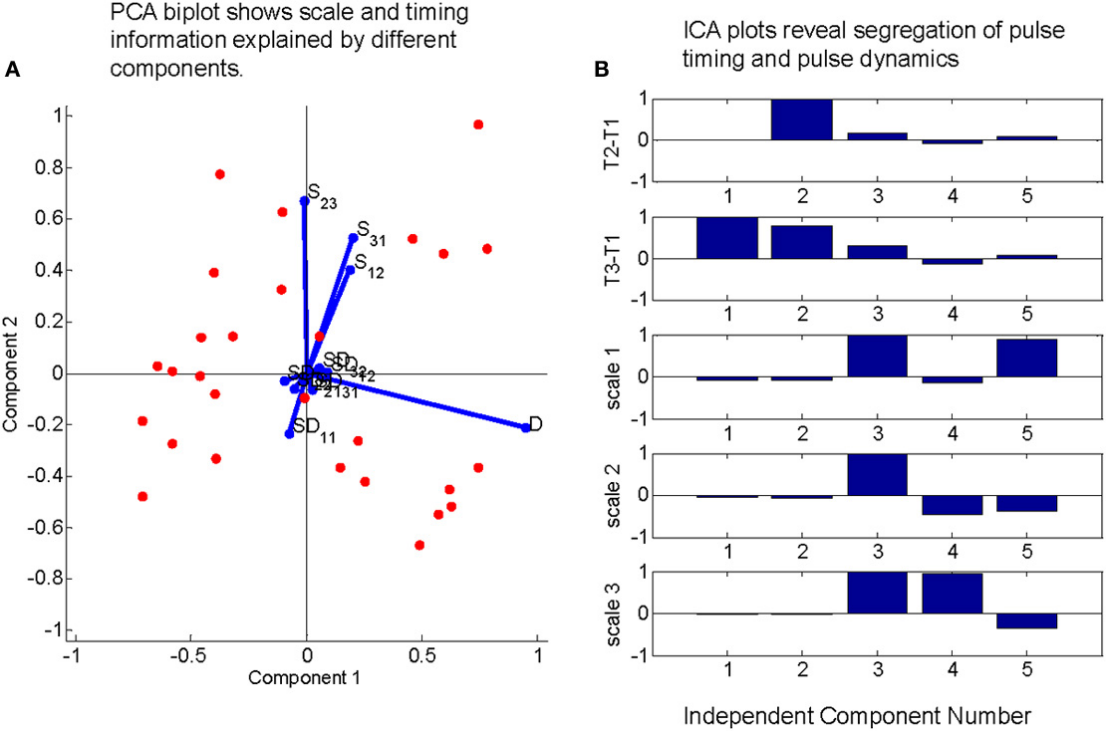
**Direct tests of whether pulse durations and pulse intervals are independent.** PCA on regressions between inter pulse delays and pulse duration time scales and ICA of delays and timescales together do not support a time-varying synergy model for real frog data. **(A)** Regression coefficient between pulse widths (D) and those between pulse time delays (S12, S31, and S23) in triplets of active motor primitives are large relative to cross terms. Additionally, these large terms align with axes defined by two largest principal components. **(B)** Mixing matrix coefficients from an independent component analysis on time series defined by each peak time difference from first element in each triplet concatenated to the scale of the pulses during counting interval demonstrate a strong segregation of time-scale related information and peak time related information. Taken together, these results indicate that the duration of pulses in the EMG do not scale linearly with the variations in the interval to their time of occurrence, which is a prediction of a TVS model.

TVS models predict a certain degree of covariance between the time scale of individual EMG activation and the relative timing of pulses in a synergy (d'Avella and Bizzi, 2005; d'Avella et al., 2006). Therefore, separate from the Q statistic analysis, we also chose to examine the functional dependence of primitive duration on the time delay between primitives. We explored this in wipes with a three primitive sequence, largely following work on triphasic bursts in human reaching (Gottlieb, 1998). We wanted to examine the degree to which primitive duration varied within these three primitive sequences, as well the dependency of primitive duration on pulse timing within a short sequence of primitive activity. Occurrences for each triplet were shuffled and significant three primitive sequences in the original data were identified by finding those triplets with z-scores greater than 2 (based on the statistics of the shuffled data). For each occurrence of a significant triplet, we calculated regression coefficients between (a) the durations of pulse 1 (D1) and pulse 2 (D2), (b) pulse 2 and pulse 3 (D3), (c) time of pulse 2-time of pulse 1 (t2-t1) and the time of pulse 3—the time of pulse 1 (t3-t1), (d) all cross terms (i.e., pulse duration 1 vs. time delay between pulse 1 and pulse 2). A principal components analysis was performed on the calculated regression coefficients, and the first two principal components were retained and plotted against one another (Figure 7A). Each of the regression coefficients was plotted on these axes as well. The regression coefficients between pulse widths (a, b) tended to align with the second principal component (Figure 7A, red lines) while the regression coefficients between pulse time delays (c) aligned strongly with the first principal component. Cross term regression coefficients tended to be smaller than either of these and were distributed more or less equally between the principal component axes.

As a second test, for each occurrence of each significant three primitive sequence, we placed the t2-t1 values for each word occurrence in one row of a multidimensional array, t3-t1 in another row, and D1, D2, and D3 in the final three rows. The resulting multidimensional array was treated as a time series and decomposed using an independent components analysis (ICA). The resultant mixing weights (Figure 7B) show a clear segregation between the time scale components (the final three components that project to D1, D2, D3) and the pulse occurrence components (the first two components which project to the t2-t1 and t3-t1 series.

### Cyclic patterns—SS and TVS compared in rat treadmill walking

**Figure 8:**
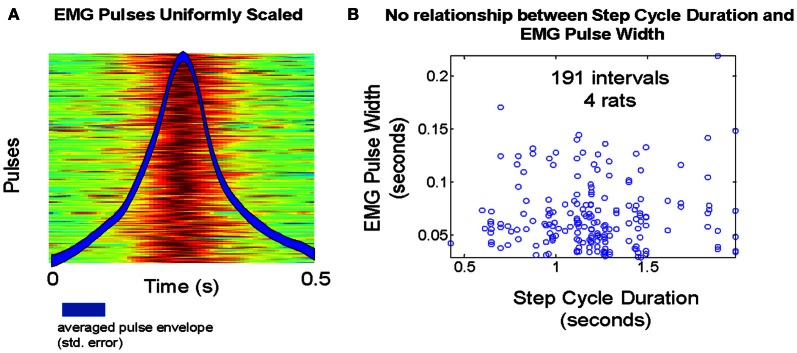
**(A)** Temperature plot of pulse amplitudes during treadmill walking in one animal. Overlay: average pulse waveform over all steps. **(B)** Step cycle duration variability does not appear to induce any systematic variability in EMG pulse full width at half max, as a TVS model predicts.

We examined the behavior of the Q statistic and related measures in rat ambulation, where cyclic patterns occur. The value of the Q statistic would be very limited if use was confined only to non-rhythmic motor behaviors. Surrogate TVS and SS data sets were constructed as in the methods. Calculation of the Q statistic was performed as described in the methods. We limited the counting time window to 400 ms in order to better deal with the compressed time scale of pulse activation observed in EMGs recorded from treadmill walking rats. Pulse widths extracted from data at a mean step rate of 1.0 step/cycle did not appear to exhibit significant variability (Figure 8A), an observation that was confirmed when we compared pulse widths against the actual step cycle length (Figure 8B).

**Figure 9:**
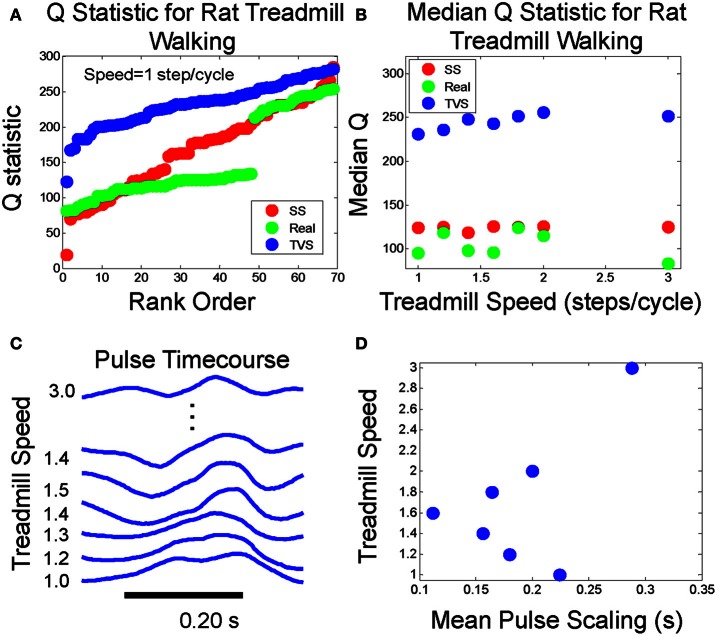
**(A)** Q statistic computed on rat walking on treadmill at 1 step/cycle compared to Q statistics for cyclic runs of SS and TVS model. **(B)** Median Q statistics for real, SS, and TVS models as a function of treadmill speed. **(C)** Example pulse waveforms at each treadmill speed. **(D)** Mean pulse duration scaling relationship with increasing treadmill speed is non-monotonic.

We then sought to systematically vary step cycle duration and observe the effect on the Q statistic and pulse scaling (Figures 9A,B). We noted that the distribution of Q values did not exhibit any noticeable trend as step cycle duration was varied, either in TVS and SS models, or in real data (Figure 9A). The Kolmogorov Smirnov test showed the rat interval structure deviated from TVS significantly (*p* < 2.3 E-15) and also from expected SS (*p* < 5.2 E-11). However, once again, as in the frog data, for the rat, the real data is found ***beyond*** the model SS cumulative curve, rather than lying between the SS and TVS curves. The discontinuity observed in the real rat EMG data Q statistic curve in Figure 9 is due to overlapping occurrence of two nearly-simultaneous, but apparently distinct, muscle groups on some trials. Because of these short-interval follow-on synergies in the rat data, the Q statistic could not go much above a certain value on the intervals where these overlaps occurred, because the short time separation between them lowered Q significantly and thus created the low values and the discontinuity seen in the Q value population rank order. This was a situation not explicitly modeled in either the TVS or SS cyclic model data. However, it does not appear to affect our results discrimination using median values (Figure 9B) but may account for the *increased* distance of rat data from TVS curves. Examining pulse time course as a function of step cycle duration (Figures 9C,D) we observed that there does not appear to be a strong monotonic trend relating cycle duration and the scaling of pulse widths. Q statistics of the real data favored the SS models over TVS patterns, even in these cyclic repeating “pattern generator” data, consistent with separation of pattern formation and rhythm generation (Rybak et al., 2006; McCrea and Rybak, 2007, 2008).

## Discussion

There is significant controversy in motor control as to the nature and origin of the strategy used for reduction of dimensionality. Many kinds of primitive—synchronous synergy, or TVS or motor pattern “block,” or kinematic stroke—have been advanced as the fundamental organizational units for the great bulk of motor activities. To date it has proven difficult to identify which organizational strategy is used in the assembly of a given motor pattern observed during behavior.

Prior work has used onset or peak timings in EMG to explicitly cluster patterns into SS (Krouchev et al., 2006; Drew et al., 2008; Markin et al., 2012). We here presented a newly developed set of tests, that are capable of discriminating presence of TVS and SS based on onset or peak timing statistics prior to any explicit clustering of data. In part, the largest value of our method is that it is applicable to the EMG time series prior to any synergy extraction or fitting processes and free of assumptions about the precise type or numbers of synergies. After this new analysis, other synergy extraction procedures, guided by the discriminant results could be applied to extract the synergy structures with better precision. We investigated the statistical properties of a range of TVS and SS models for the construction of muscle activity and found that the Q statistic we developed represented a good discriminant over a large range of parameters when applied to the EMG time series in this way.

### Range of applicability of Q

For a large range of parameters, it is possible to discriminate models arising from a TVS model from those arising out of a SS model by examining the EMG (or mixed) output time series' Q statistic. We varied three parameters in modeling phase of this study*:* the density of synergies occurring on an interval (ρ), the number of muscles per synergy (σ) and the number of simultaneous muscles in a TVS model (η). In general, the models became harder to discriminate as η increased (i.e., for mixed models), and we also observed that varying the shape of the synergy membership (σ) distribution appeared to have the strongest effect on the significance of these discriminations.

These limitations on results are not surprising: If most primitives have between 2 and 4 synchronous muscles active during their activation (as is the case in the peaked distribution) it will become much more difficult to discriminate TVS constructed data from SS data as the number of synchronous muscles mixed into the set of time-varying synergies increases. In effect, the time-varying synergies look more and more like SS in this case, because of the larger number of small delays. However, we found that in real data sets from rats and frogs the Q statistic we found was unambiguous, and was structurally always clearly in the SS model domain. In fact the real data Q statistic curve was “more” SS (i.e., further from the TVS curve) than the randomly generated family of SS data curves, and in both instances (frog and rat) the data lay further beyond these SS curves rather than between SS and TVS. The Q statistic of actual real world motor patterns and synergy data is a specific realization of one of the SS family of realizations and apparently strongly different.

As a test of the generalizability of this approach, we did the analysis of cyclic data using treadmill walking data from adult rats. The behavior of the Q statistic was qualitatively the same, although some adjustment had to be made for the fact that bursts of activity in rat EMG tend toward shorter time scales in the rat, at least during treadmill walking behaviors, and short latency synergy burst overlaps occurred in some cycles. Our Q statistic results for the rat were consistent with the idea of separation of rhythm and pattern components of CPG output suggested by McCrea and Rybak. The Kolmogorov Smirnov test shows the rat interval structure deviates from TVS significantly (*p* < 2.3 E-15) and also from expected SS (*p* < 5.2 E-11). However, the Q aligns well with SS Q behavior over much of its range (Figure 9A). We attribute the deviation in mid range to the cyclical pattern of locomotion, occasional closely overlapped or near synchrony of specific synergies during the cycle, and consequent deviations from the levels of intermittency used in the original simulations of motor patterns generating the expected Q distributions. A more complete exploration of the Q statistic and other interval-pulse-scaling metrics in the cyclic patterns may provide significant insight into the mechanisms underlying locomotor flexibility in quadrupedal mammals.

### What Q tells us about synergies in the frog

We compared SS to time-varying synergies using a linear discriminant analysis with the Q statistic. Differences were taken between Q value from each model and Q values computed on real data. These differences were then plotted against one another and a separatrix representing equal differences was used as a criterion for classification. Those “below” the line represented values where Qtvs-Qreal was greater than Qss-Qreal. I.E, i.e., these points were “closer” to a SS model than a TVS model. The separatrix used in this case was thus a line with slope of 1.

A broadly similar pattern was seen to that in the preceding section. Discrimination was possible for synergies with muscle densities (σ) drawn from a flat distribution, but became more difficult for σ drawn from the peaked distribution as η (number of simultaneous muscles in a synergy) was increased. The results for comparison of frog data to a flat σ distribution are unambiguously indicative of an SS model. Assuming instead that the σ is from a peaked distribution in frogs, it is still most likely the case that synergies observed in the bullfrog during a variety of reflex and locomotor tasks arise only from SS. The only alternative would be very weak scaling of time-varying and very high jitter synergies, within which most muscles are activated synchronously. This alternative may beg the question, and weakens the TVS formulation elegance and simplicity of more strongly coupled units. It also may not match other measures of synergy compositionality. The real data lay outside the bounds of both the TVS and SS curves, below the SS curves in both rat and frog. In the rat, inspection of a discontinuity revealed an intermittent coactivation of two synergies caused the discontinuous and outward deviation. Similar coactivations occur in frogs (e.g., in wiping behaviors). The statistics associated with these tight coactivations or “synergies of synergies” were not well represented in our simulations and would have been very rare in our generator processes. However, the processes noted would further differentiate the point process statistics between TVS and SS rather than collapsing them together, and should not be assumed *a priori*.

As a final check on the discrimination we used an interesting side effect of the Q statistic that we discovered, namely that rescaling the pulse times, thereby moving some pulses outside the bounds of the 250 ms counting interval does not appreciably change the TVS Q statistic, but significantly alters the SS Q statistic. We used this as an additional check to ascertain whether TVS or SS models statistics better explain observed EMG data from the frog. Because the statistic is only sensitive to rescaling within each counting intervals, rescaling the time differences between nearly SS results in large changes to the Q statistic relative to the unscaled data. These increases in ΔQ occur with increasing scaling, until plateauing at a particular Δ Q value (the point at which “rescaled” pulses are pushed into the next counting window). Rescaling TVS (time-varying synergies) fails to alter ΔQ appreciably for TVS models. Consonant with the other results so far, performing these operations on real EMG data we found that the data rescaled as one would expect SS to scale. The coactive synergies noted above in real frog and rat data would likely have further exacerbated these scaling effects.

Taken together, these data and analyses lead to the following conclusion: at least in the bullfrog, and SS model, or synergist coactivation of groups of muscles appears to be the norm. Any strategies similar to the TVS model are much more of an exception, at least within this preparation.

#### Covariance of muscle activation and pulse timing

As a final check for any evidence of time-varying synergies in the frog, given the Q statistic results, we explored directly whether the time differences between pulses in a three pulse triplet depended at all on the pulse widths in that sequence. This would indicate correlated time-rescaling. PCA performed on regression coefficients between pulse time differences and time widths found that the most significant contributions to variance were entirely from components closely aligned to either the time difference axes, or pulse width axes. In contrast, PCs representing regression cross terms between pulse widths and pulse time differences contributed to very much less variance to the overall data set. The results thus indicate that there is little overlap or covariation between pulse width and pulse time/phasing differences and that the bulk of the variation of the delay between pulses in the EMG is independent of the variations underlying pulses durations. This observation is inconsistent with the notion of a uniformly scaled TVS, but matches the Q statistic data presented here. In frogs other data support SS structure based on ICA decomposition (Hart and Giszter, 2004), neural analyses (Hart and Giszter, 2010) and explicit physiological perturbations. We have demonstrated the ability to recruit SS synergies as single pulses (Kargo and Giszter, 2000a,b), and perturb them separately within a motor pattern (Kargo and Giszter, 2008), both inconsistent with TVS descriptions.

To bolster the temporal structure observations here, we performed an ICA on all time differences in the data set, and the corresponding pulse widths. We found that the resulting mixing matrixes indicated very little mixing between pulse width and time differences, with components contributing primarily to one or the other category of time series. This observation is also inconsistent with uniformly or correlated scaling of pulse duration and sequence as expected in TVS. The rat cyclic data also showed a lack of correlation between pulse duration and cycle duration in our data.

In summary, we present a new analysis working directly with EMG peak or onset data to differentiate synchronous and TVS patterns prior to full decomposition. These analyses applied to data from frog and rat support composition of frog and rat motor patterns as independent rhythms and synchronous synergy pulses, consistent with a separated control of the rhythm/phase and pattern compositional elements. These separated controls may be important to allow the force/effector compositional controls to adjust as needed to support the next level of task composition. Unitary task elements at the kinematic and kinetic levels of task description occur in reaching, and locomotion, but adaptation of these to momentary conditions may require less stereotypy in the supporting compositionality of muscle synergy bursts and pattern.

### Conflict of interest statement

The authors declare that the research was conducted in the absence of any commercial or financial relationships that could be construed as a potential conflict of interest.
